# Proteomic identification of early urinary-biomarkers of acute kidney injury in preterm infants

**DOI:** 10.1038/s41598-020-60890-x

**Published:** 2020-03-04

**Authors:** Young Hwa Jung, Dohyun Han, Seung Han Shin, Ee-Kyung Kim, Han-Suk Kim

**Affiliations:** 1 Department of Pediatrics, Seoul National University Children’s Hospital, Seoul, South Korea; 2 Department of Pediatrics, Seoul National University College of Medicine, Seoul, South Korea; 3 Department of Pediatrics, Seoul National University Bundang Hospital, Seongnam, South Korea; 4 Proteomics core facility, Biomedical Research Institute, Seoul National University Hospital, Seoul, South Korea

**Keywords:** Biomarkers, Paediatric research, Kidney diseases, Proteomic analysis

## Abstract

The immature preterm kidney is likely to be vulnerable to acute kidney injury (AKI). However, the biomarkers currently used for AKI are not sensitive or specific and are also inadequate for the timely detection of AKI in preterm infants. The objectives of this study were to identify novel urinary biomarkers of AKI using proteomic techniques, and to verify and validate that the candidates can serve as early predictive biomarkers for AKI. In total, 1,810 proteins were identified in the discovery phase. Among those proteins, 174 were selected as the 1^st^ targeted proteins. A total of 168 proteins were quantified, and the levels of 6 were significantly increased in the AKI group in the verification phase. Using a clinical assay, the results were confirmed and validated using samples of the first urine after birth from the biorepository. Finally, enzyme-linked immunosorbent assays revealed that the levels of annexin A5, neutrophil gelatinase-associated lipocalin (NGAL), and protein S100-P were significantly higher in the samples of the first urine from patients with AKI than in those from patients without AKI. In conclusion, urinary annexin A5, NGAL and protein S100-P levels are promising biomarkers for early, accurate prediction of AKI in preterm infants.

## Introduction

Although data from neonatal acute kidney injury (AKI) research has been sparse until recently, previous epidemiological studies have suggested that AKI is common in neonates, and those with AKI are at risk for death and long-term chronic kidney disease^1,2^. Several single-center studies showed that the incidence of AKI in very-low-birth-weight infants was 15% to 40%, and the mortality was significantly higher in preterm infants with AKI than in those without AKI^3–5^. However, the incidence may be underestimated because of poor detection of nonoliguric renal failure in preterm infants and the serum creatinine (SCr)-based definition of AKI.

AKI is usually diagnosed indirectly by a measured increase in the level of SCr, which is thought to indicate a reduction in the glomerular filtration rate (GFR). However, creatinine is an unreliable indicator during acute changes in kidney function because changes in the SCr levels are a late consequence of kidney damage. SCr concentrations can vary widely based on age, sex, muscle mass, muscle metabolism, and medications^6^. In neonates, several endogenous and exogenous factors could contribute to the delay in diagnosis of AKI based on SCr^7^. Neonatal SCr levels during the first few days of life may not reflect their own kidney function but rather the mother’s kidney function via the placenta. In addition, normal ranges of SCr change over time depending on gestational age and postnatal age^8,9^. SCr concentrations usually fluctuate significantly over the first weeks of a newborn’s life^10^. In preterm infants in particular, there is a very wide distribution of normal SCr values that change over time, depending on the degree of prematurity^8^. In addition, measuring SCr frequently in small infants is not advised because of a concern about the amount of blood loss due to multiple blood samplings. Regardless of these limitations, SCr still remains the standard tool used for the diagnosis of AKI because of the lack of appropriate biomarkers for AKI in neonates.

Recently, the application of innovative technologies, such as functional genomics and proteomics, has revealed several novel genes and gene products that are emerging as biomarkers. In contrast to the genome, which is unique and relatively stable, proteomes are cell- and tissue-specific and change over time in direct response to different situations. Typically, tissue analysis involves genomic approaches, whereas body fluids are best analyzed by proteomic techniques. Therefore, we considered that proteomics is a promising tool with which to search for biomarkers that directly reflect the pathological status in biological fluids^11^. Among the biological fluids, urine is most likely to contain biomarkers arising from the kidney. Urine is easily accessible in a large quantity without the use of invasive procedures and reflects the pathophysiological changes in the genitourinary tract and kidneys. Urinary proteins have been shown to remain stable long enough to enable performance of reliable proteomic analysis^12^.

The objectives of this study were 1) to identify novel urinary biomarkers of AKI using proteomic techniques, and 2) to verify and validate that the discovered candidates are early predictive biomarkers for AKI in extremely preterm infants.

## Results

### Discovery phase

**Figure 1:**
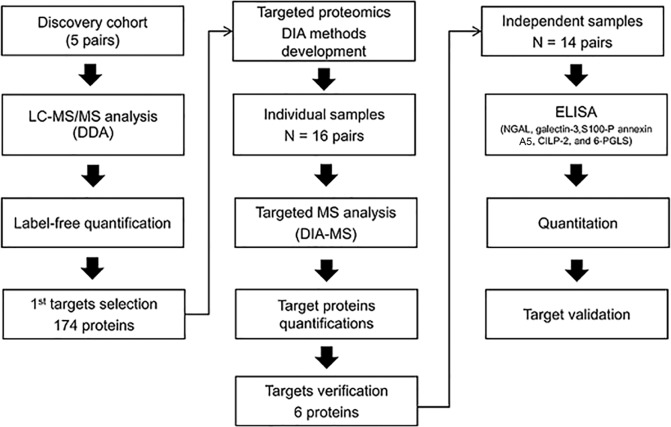
Work flow in proteomic biomarker discovery experiments. LC MS/MS, liquid chromatography-tandem mass spectrometry; DDA, data-dependent acquisition; DIA, data-independent acquisition; ELISA, enzyme-linked immunosorbent assays.

**Figure 2:**
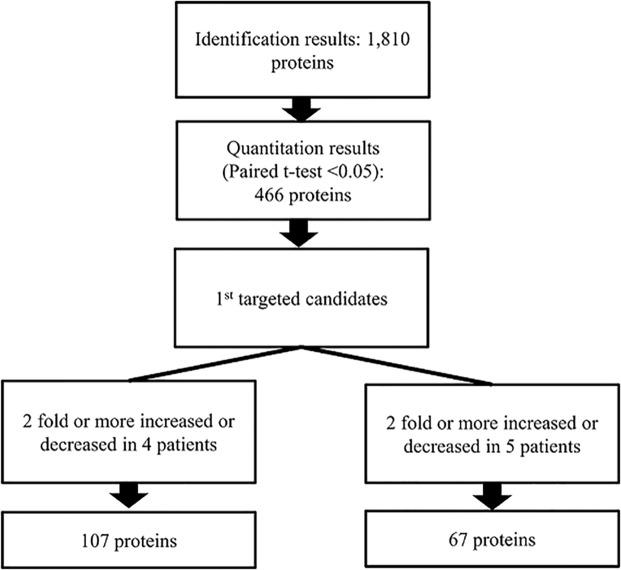
Schematic representation of the target protein selection process in the discovery phase.

Among the 37 infants who were enrolled in the cohort study, 5 patients were identified as having AKI after ibuprofen treatment. The multiquantitative proteomics approach depicted in Fig. 1 was used to identify urinary proteins differentially expressed before and after ibuprofen administration in patients with AKI induced by ibuprofen treatment. In total, 1,810 proteins were identified (Supplementary Table S1), the distributions of the identified proteins according to the involved cellular components and biological processes are shown in Supplementary Figs. 1 and 2. Among them, 349 unique proteins showed levels that differed by two-fold or more, with 107 changed in 4 patients and 67 changed in 5 patients. We selected 174 proteins that were either up- or down-regulated in AKI patients as the 1^st^ targeted candidates: 55 up-regulated proteins and 52 down-regulated proteins in 4 patients, and 36 up-regulated proteins, and 31 down-regulated proteins in 5 patients were included (Fig. 2 and Supplementary Table S2).

### Verification phase

From the biorepository, sixteen urine samples from patients with AKI and 16 samples from controls that were matched by gestational age and postnatal age in the biorepository were selected and analyzed. We performed data-independent acquisition (DIA)-based targeted proteomics using Spectronaut software. We were able to quantify 168 of the 174 proteins identified in the discovery phase (Supplementary Table S3). Among them, the levels of only six proteins were significantly different, and these proteins were all up-regulated in the AKI group compared to the control group. Neutrophil gelatinase-associated lipocalin (NGAL), cartilage intermediate layer protein 2 (CILP-2), 6-phosphogluconolactonase (6-PGLS), annexin A5, galectin 3, and protein S100-P were finally selected as final candidate urinary AKI biomarkers.

### Validation phase

To evaluate the predictive value of the 6 urinary biomarkers for AKI, we selected additional patients who developed AKI within 7 days after birth without any postnatal insults and those who did not develop AKI within 7 days after birth for the control group from the repository and matched them according to gestational age. Samples of the first urine collected within 24 hours after birth were also analyzed. Fourteen infants who were identified as having AKI within 7 days after birth and 14 infants matched by gestational age without AKI were selected. Prenatal, clinical, and laboratory characteristics are listed in Table 1. Infants with AKI had lower 1- and 5-minute Apgar scores. There were no differences in the perinatal characteristics, including preeclampsia and oligohydramnios. Of those with AKI, 4 of 14 (28.6%) died, compared with 1 of 14 (7.1%) of those without AKI. In the AKI group, 6 patients developed chronic kidney disease after discharge.

**Table 1:** Basal characteristics for the validation set.

	AKI (n = 14)	No AKI (n = 14)	p-value
**Patient demographics**			
Gestational age (week), mean ± SD	27.6 ± 2.6	27.9 ± 2.6	0.8
Birth weight (g), mean ± SD	905.1 ± 399.1	1070.0 ± 296.5	0.282
Male, n (%)	5 (35.7)	7 (50)	0.704
Small for gestation (<10 percentile), n (%)	3 (	1 (7.1)	0.596
Apgar score at 1 minute, median (min, max)	2 (0–6)	4 (1–7)	0.016
Apgar score at 5 minutes, median (min, max)	5 (2–7)	7 (5–10)	0.004
Mortality, n (%)	4 (28.6)	1 (7.1)	0.326
Chronic kidney disease, n (%)	6 (42.9)	1 (7.1)	0.077
**Prenatal characteristics**			
Antenatal steroid, n (%)	8 (57.1)	10 (71.4)	0.43
Maternal preeclampsia, n (%)	0 (0)	1 (7.1)	1
PROM, n (%)	6 (42.9)	7 (50)	0.705
Histologic chorioamnionitis, n (%)	6 (42.9)	5 (35.7)	0.699
Maternal kidney disease, n (%)	0 (0)	1 (7.1)	1
Oligohydramnios, n (%)	4 (28.6)	0 (0)	0.098
Diabetes mellitus, n (%)	0 (0)	0 (0)	1
**Laboratory measurements (within 7 days)**			
SCr at cord blood (mg/dL), mean ± SD	0.38 ± 0.18	0.49 ± 0.17	0.089
SCr at PND 3 days (mg/dL), mean ± SD	1.64 ± 0.59	0.77 ± 0.14	<0.001
SCr at PND 7 days (mg/dL), mean ± SD	2.05 ± 0.55	0.67 ± 0.18	<0.001
Δ SCr between PND 3 days and 7 days (mg/dL), mean ± SD	0.41 ± 0.36	−0.09 ± 0.13	<0.001

AKI, acute kidney injury; PROM, premature rupture of membrane; SCr, serum creatinine; PND, postnatal day.

**Figure 3:**
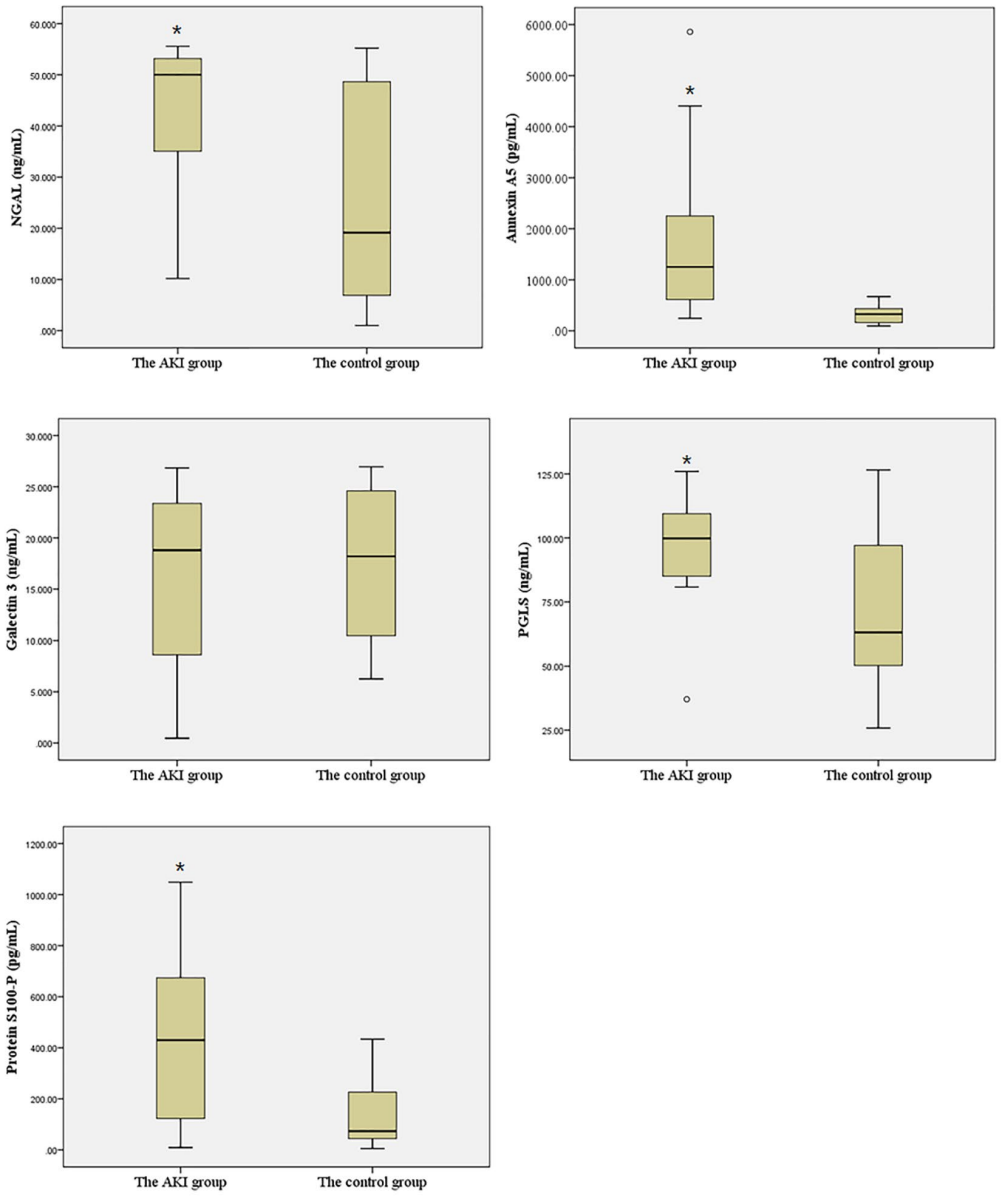
Box and whisker plots of urine values of NGAL, annexin A5, S100P, 6-PGLS, and galectin 3 in preterm infants with AKI and infants without AKI during the first day of life. *p-value <0.05, data are presented as box and whisker plots NGAL, neutrophil gelatinase-associated lipocalin; 6-PGLS, 6-phosphogluconolactonase; AKI, acute kidney injury.

Six final candidate urinary biomarkers were measured with enzyme-linked immunosorbent assays (ELISAs) in 28 samples of the first urine. The levels of each biomarker were compared between infants with AKI and those without AKI, as shown in Table 2. Among the 6 urinary biomarkers, CILP-2 was not compared between the AKI patients and controls because of the very low levels in both groups. NGAL was significantly elevated in the AKI group compared with that in the control group (44.53 ng/mL, 95% confidence interval (CI) 34.52–51.41 vs. 25.18 ng/mL, 95% CI 11.16–38.02; p = 0.008). Similarly, annexin A5 (1877.11 pg/mL, 95% CI 689.43–2874.79 vs. 320.65 pg/mL, 95% CI 232.13–457.86; p = 0.006), 6-PGLS (95.81 ng/mL, 95% CI 79.32–105.20 vs. 70.39 ng/mL, 95% CI 47.63–92.56; p = 0.023), and protein S100-P (434.88 pg/mL, 95% CI 187.44–674.55 vs. 146.86 pg/mL, 95% CI 50.80–242.93; p = 0.019) were significantly elevated in the AKI group compared with their levels in the control group (Fig. 3).

**Table 2:** Urinary biomarkers according to acute kidney injury status.

Protein	AKI (n = 14)	Control (n = 14)	p-value	AUC
NGAL, ng/mL	44.53 (34.52, 51.41)	25.18 (11.16, 38.02)	0.008	0.75
Annexin A5, pg/mL	1877.11 (689.43, 2874.79)	320.65 (232.13, 457.86)	0.006	0.882
Galectin 3, ng/mL	16.49 (9.30, 20.84)	16.77 (12.26, 21.06)	0.929	0.472
6-PGLS, ng/mL	96.81 (79.32, 105.20)	70.39 (47.63, 92.56)	0.023	0.667
Protein S100-P, pg/mL	434.88 (187.44, 674.55)	146.86 (50.80, 242.93)	0.019	0.748

NGAL, neutrophil gelatinase-associated lipocalin; 6-PGLS, 6-phosphogluconolactonase; AKI, acute kidney injury.

For every 10 ng/mL increase in the concentration of NGAL, the odds of having AKI increased by 79% (OR = 1.79, 95% CI 1.12–3.05; p = 0.018). For the same increase in the concentration of PGLS, the odds increased by 38% (OR = 1.38, 95% CI 1.02–1.89; p = 0.035). For every 100 pg/mL increase in the level of annexin A5, the odds of having AKI increased by 82% (OR = 1.82, 95% CI 1.11–2.99; p = 0.031).

**Figure 4:**
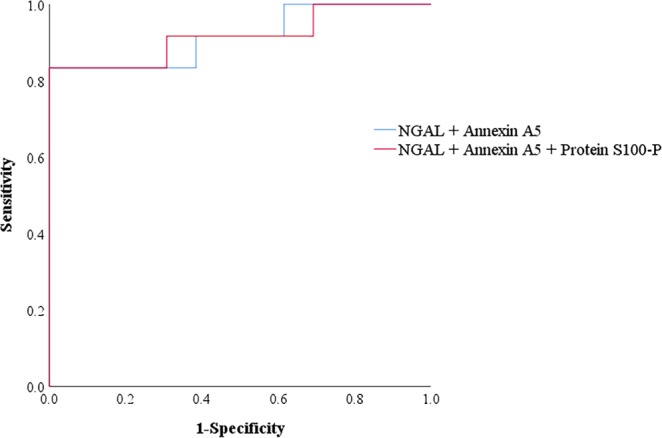
ROC AUC values for combined urinary biomarkers; their predictive abilities to detect AKI in preterm infants. ROC, receiver operating characteristic; AUC, area under the curve; NGAL, neutrophil gelatinase-associated lipocalin; AKI, acute kidney injury.

The maximum values of these biomarkers were well able to predict AKI; the areas under the curve (AUCs) were 0.75 for NGAL, 0.882 for annexin A5, 0.748 for protein S100-P and 0.667 for 6-PGLS (Table 2). Combining NGAL and annexin A5 in one model improved the ability to detect AKI (AUC = 0.923). Combining NGAL, annexin A5, and protein S100-P increased the AUC value to 0.932 (Fig. 4).

The derived sensitivities, specificities, and predictive values at the determined cut-off concentrations for the urinary biomarkers are listed in Table 3.

**Table 3:** Urinary biomarker characteristics for prediction of AKI.

Biomarker	Cut-off	Sensitivity	Specificity	PPV	NPV
NGAL (ng/mL)	42.15	0.71	0.71	0.71	0.71
Annexin A5 (pg/mL)	409.86	0.85	0.71	0.73	0.83
Protein S100-P (pg/mL)	110.70	0.83	0.64	0.77	0.78

NGAL, neutrophil gelatinase-associated lipocalin; PPV, positive predictive value; NPV, negative predictive value.

## Discussion

To the best of our knowledge, this is the first proteomic study to identify novel urinary AKI biomarkers in preterm infants.

This study described the identification, verification and validation of urinary biomarkers for AKI in extremely preterm infants. NGAL, annexin A5, and protein S100-P showed excellent abilities to differentiate between patients who developed AKI within 7 days after birth and those who did not. We used samples of the first urine, which were collected within 24 hours after birth, and these biomarkers showed good prognostic potential, with increased concentrations of the biomarkers associated with the development of AKI. When the accuracy was assessed using receiver operating characteristic (ROC) curves, the combination of NGAL and annexin A5, as well as NGAL, annexin A5, and protein S100-P, performed well.

Urine NGAL is postulated to be a highly sensitive marker of AKI, specifically of tubular cell damage rather than a decrease in glomerular filtration^13^. Urinary NGAL levels are strongly correlated with SCr levels, and this identification preceded that of traditional markers in critically ill pediatric and adult populations. However, only a few studies have analyzed urine NGAL levels in preterm infants. In one such study, urine NGAL concentrations in preterm infants showed low sensitivity for the detection of renal impairment and appeared variable in preterm populations^10^. Suchojad *et al*. also demonstrated that inflammatory status and immaturity limit the specificity of urine NGAL as a marker of AKI^14^. In contrast, we showed that urine NGAL levels obtained during the first day of life can predict AKI with a sensitivity and specificity of 0.71.

There is a lack of data supporting the use of S100P and annexin A5 as biomarkers for predicting AKI. However, many vital physiological functions and metabolic processes are regulated by Ca^2+^, and a large number of human diseases are linked to altered Ca^2+^ homeostasis. Ca^2+^ signals within cells are transmitted by many Ca^2+^ binding proteins, including the S100 protein family and annexins.

The S100 proteins are the largest subgroup within the superfamily of EF-hand Ca^2+^ -binding proteins. S100 proteins have been investigated because of their close associations with human disease, including cancer, neurodegenerative disorders, and cardiomyopathies, and their recent use in diagnosing disease. S100P, a member of the S100 protein family, was initially identified in the placenta, and its widespread expression was later noted in the gastrointestinal tract, prostate, leukocytes, and renal pelvic urothelium^15^. S100P is known to be expressed in several malignant neoplasms, especially renal neoplasms^16^. Although we identified that S100P could be a novel biomarker for AKI in the present study, there have been no previous studies on the role and expression of S100P during the development of the kidneys during the fetal period. Therefore, further studies are needed to investigate the association between S100P and kidney injury, including underlying biological pathophysiology.

Annexins are also Ca^2+^ and phospholipid-binding proteins forming an evolutionarily conserved multigene family. Some annexins have been considered to participate in the regulation of membrane organization and trafficking, as well as the regulation of ion currents across membranes^17,18^. In addition, some members of this family have been identified as receptors for endothelial proteases and inhibitors of neutrophil migration and blood coagulation. Deregulation of annexin expression and activity, which might have stress related functions, has been associated with human disease. Among the annexin family, Annexin A5 has been found to be upregulated by changes in the cellular hypoxic state, and identified as a marker of apoptotic changes induced by hypoxic stress^19–21^. As ischemic renal dysfunction significantly contributes to apoptosis, annexin A5 can be a biomarker for predicting AKI. Regarding the lack of studies on these biomarkers, which were first discovered in the present study, further studies would be helpful for understanding the precise functions and pathophysiological mechanisms of individual biomarkers.

To date, several studies on biomarkers of kidney injury have investigated multiple markers capable of the early detection of structural injury that is also associated with outcomes. There are a few promising urinary biomarkers, such as NGAL, IL-18, and KIM-1, that can be representative sequential biomarkers that would be useful for determining the timing of the initial insult and assessing the duration of AKI^22^. Although these results are promising, individual biomarkers still lack sensitivity and specificity. Biomarkers with an AUC value of 0.5 are not better than expected by random chance, and a biomarker with an AUC of approximately 0.75 is generally considered to be a good biomarker. In this study, we demonstrated that the AUC value for combining urinary NGAL, S100P, and annexin A5 was 0.932, which represents an excellent biomarker.

This study has several strengths. First, we conducted a prospective cohort study, which allowed the data to be collected consistently, and all factors were taken into account in the study design and the data analysis and interpretation. Different samples were used in each stage, and the candidate biomarkers were ultimately confirmed using conventional immunoassays. Second, several previous studies have shown that the urinary concentrations of some biomarkers are dependent on gestational age in neonates without AKI^23^. This may be secondary to the inability of immature tubules to reabsorb these proteins in underdeveloped kidneys. Therefore, controlling for gestational age is necessary when determining the associations between urinary biomarkers and AKI. In this study, we selected urine samples from gestational age- and postnatal age-matched neonates for the control group. Third, we did not use urine creatinine concentration because this can also reflect kidney injury. Instead of calibrating the urinary creatinine level in all urine samples, the amounts of protein in each sample were adjusted to the same value in this study^24^. Forth, for identification and verification of urinary biomarkers for AKI, we analyzed urine samples that met the definition of AKI as an increase in SCr. Instead, when validating the biomarkers, we used samples of the first urine before the increase in SCr, which likely reflects prenatal kidney function in preterm infants. Premature infants have with a very low glomerular filtration rate; therefore, mild exposure to nephrotoxic agents can cause a high degree of injury^25^. However, premature infants are at significantly elevated risks for sepsis and exposure to nephrotoxic medications such as antibiotics and COX inhibitors. Additionally, most preterm infants who develop AKI are extremely premature and develop AKI within the first week after birth^3^. Of note, neonatal serum biomarkers such as Cr during the first few days of life may reflect the mother’s kidney function but not their own. The advantage of using the first urine after birth is that we can directly evaluate the potential risk of AKI by estimating underlying prenatal kidney insults induced by prenatal pathological conditions and perinatal insults and can ameliorate AKI by preventing modifiable risk factors.

This study has some possible limitations because of the small sample size. After the discovery and initial validation of biomarkers, further large-scale studies are needed to confirm their clinical utility. With regard to the clinical application of these urinary biomarkers, the estimation of the amount of damage for risk stratification and prediction of disease progression and clinical outcomes should also be performed.

In conclusion, this study provides new insights into the potential role of proteomics in the identification of novel urinary AKI biomarkers in premature infants. According to our results, NGAL, S100P, and annexin A5 can predict the risk of AKI at the time of birth. In addition, the combinations of NGAL, S100P, and annexin A5 significantly improved the ability to predict AKI. The early detection of kidney injury can be helpful clinically for neonatologists in the prevention of further postnatal kidney insults.

## Methods

### Study design

The biomarker development process was divided into the following 3 phases: the discovery, verification, and validation phases.

### Discovery phase

We conducted a prospective cohort study at Seoul National University Children’s Hospital between 2015 and 2017 that was designed to enroll preterm infants with birth weights <1,500 g or gestational ages <32 weeks who were treated with ibuprofen for patent ductus arteriosus. We collected serial urine samples during the 6 hours before starting ibuprofen treatment and after ibuprofen treatment. The SCr concentration was measured as part of routine preterm care. These data were used to select patients who developed AKI after ibuprofen treatment. To identify protein biomarker candidates in the discovery cohort, we performed label-free mass spectrometry (MS)-based protein quantification with 10 urine samples.

### Verification phase

The biospecimen archive has been maintained by the neonatal intensive care unit at Seoul National University Children’s Hospital since 2008. All preterm infants with birth weights <1,500 g or gestational ages <32 weeks who were admitted to Seoul National University Children’s Hospital were eligible. All samples were prospectively collected from the time of birth until 36 weeks postmenstrual age or hospital discharge according to the established standardized procedures for sample collection.

We conducted a nested case-control study using urine samples from the biospecimen repository to verify the identified candidates as biomarkers for predicting AKI. In this study, patients who developed AKI in the defined cohort were identified. Then, controls matched by gestational age and postnatal age were selected from the same cohort. To verify the candidate biomarkers identified in the discovery cohort, we implemented a targeted proteomics approach. In total, 36 samples were analyzed according to the DIA method.

### Validation phase

Using the biospecimen repository, we conducted another nested case-control study to validate the identified candidate biomarkers. To determine whether the candidate biomarkers can be used to detect prenatal AKI in preterm infants, we selected patients who developed AKI within 7 days after birth without any postnatal insults, such as administration of nephrotoxic medications, sepsis and hypotension. Then, for the control group, we selected those who did not develop AKI within 7 days after birth and matched them according to gestational age.

To evaluate the ability of the candidate biomarkers to predict AKI, ELISAs were performed using the first urine samples after birth. Urinary biomarkers were measured in duplicate using commercially available ELISA kits. Urinary NGAL and galectin-3 were assessed by ELISA (Quantikine, R & D systems, Minneapolis, MN), with average confidences of variability (CVs) of 2.9% and 6.4%, respectively. Protein S100-P, annexin A5, CILP-2, and 6-PGLS were measured by ELISA kits (MyBiosource, San Diego, CA, USA), with average CVs of 12.9%, 8.4%, 5.5%, and 6.2%, respectively.

### AKI definition

AKI was defined as an acute increase in the SCr of at least 0.3 mg/dL within 48 hours (stage 1 according to the AKI Network definition) or a persistent increase in SCr to ≥1.7 for 3 days after birth^7,26^. The AKI Network definition utilizes changes in serum creatinine concentration and urine output to characterize three levels of renal dysfunction. An abrupt (within 48 hours) reduction in kidney function defined as an absolute increase in serum creatinine concentration by either >0.3 mg/dL or an increase of ≥ 50% (1.5 fold from baseline) or reduction in urine output (documented oliguria of <0.5 mL/kg/hour for >6 hours). We did not use urine output criteria because premature infants often have nonoliguric renal failure due to immature tubular development. The controls were infants with at least 2 blood samples to confirm negative AKI status at the time of urine sample collection.

### Urine collection and sample preparation

Urine was collected by placing cotton balls at the perineum. The urine was extracted, centrifuged for 10 minutes to remove any cotton fibers and cellular elements, and then frozen at −70 °C until sample evaluation. The starting volumes of the infant urine samples ranged from 200 to 500 µL. After centrifugation at 3000 × g for 15 minutes at 4 °C, the urine supernatants were concentrated with an Amicon Ultra centrifugal filter device (3 kDa MWCO, Millipore) at 14,000 × g to a volume of ~50 µL. The protein content of the final concentrated solution was determined with the Bradford method (Bio-Rad Protein Assay, Bio-Rad). For the label-free quantification in the discovery phase, 50 µL of urinary proteins were precipitated by adding a 6-fold volume of ice-cold acetone prior to the digestion step. For the DIA analysis in the verification phase, 25 µL of urinary proteins per individual sample was used. The precipitated proteins were dissolved in sodium dodecyl sulfate-containing denaturation buffer. After being heated at 95 °C, the denatured proteins were digested by a filter-aided sample preparation method^27,28^. The proteins were digested with trypsin (enzyme-to-protein ratio [w/w] of 1:100) at 37 °C overnight. All samples were desalted using homemade C18-StageTips as previously described^27,28^. Then, a StageTip-based high-pH peptide fractionation was performed for the library samples in the discovery and verification phases. The desalted peptide samples and fractionated peptide samples were dried in a vacuum centrifuge and stored at −80 °C until liquid chromatography-tandem mass spectrometry (LC-MS/MS) analysis.

### LC-MS/MS analysis

All LC-MS/MS analyses, which were performed by the data-dependent acquisition (DDA) and DIA methods, were conducted with an Ultimate 3000 UHPLC system (Dionex, Sunnyvale, CA, USA) coupled to a Q-Exactive Plus mass spectrometer (Thermo) as previously described with some modifications^27,29^. Peptide samples were separated on a two-column system with a trap column and an analytical column (75 µm × 50 cm) with 120-minute gradients from 7% to 32% acetonitrile at 300 nl/minutes. The column temperature was maintained at 60 °C using a column heater. The column eluent was delivered to the Q-Exactive Plus via nanoelectrospray. In the DDA method for label-free quantification, a survey scan (350 to 1650 m/z) was acquired with a resolution of 70,000 at m/z 200. A top-20 method was used to select the precursor ion with an isolation window of 1.2 m/z. The MS/MS spectrum was acquired at an HCD-normalized collision energy of 30 with a resolution of 17,500 at m/z 200. The maximum ion injection times for the full and MS/MS scans were 20 and 100 ms, respectively. The hyper reaction monitoring (HRM) DIA method consisted of a survey scan at 35,000 resolution from 400 to 1,220 m/z (automatic gain control target of 3 × 10^6^ or 60-ms injection time). Then, 19 DIA windows were acquired at a resolution of 35,000 with an automatic gain control target of 3e6 and auto injection time^30^. The stepped collision energy was 10% at 27%.

### Data processing for label-free quantification

All MS raw files were processed by using the interface of MaxQuant (version 1.5.3.1)^31^. MS/MS spectra from the Human UniProt protein sequence data set (December 2014, 88,657 entries) were searched using the Andromeda search engine^32^. Primary searches perforemd using a 6-ppm precursor ion tolerance when total proteins were analyzed. The MS/MS ion tolerance of 20 ppm was used. Carbamido-methylation of cysteine was specified as the control modification, and N-acetylation of protein and oxidation of methionine were considered as variable modifications. Enzyme specificity was set to full tryptic digestion. Peptides with a minimum length of six amino acids and up to two missed cleavages were included. The acceptable false discovery rate (FDR) was set to 1% at the peptide, protein, and modification levels. To maximize quantification events across samples, we enabled the ‘Match between Runs’ option of the MaxQuant platform. For label-free quantification, the Intensity Based Absolute quantification (iBAQ) algorithm^29^ was used as part of the MaxQuant platform. The iBAQ values calculated by MaxQuant are the raw intensities divided by the number of theoretical observable peptides. Thus, the iBAQ values provide proportional to the molar quantities of the proteins.

### Data processing for the DIA MS

To generate the spectral libraries, 12 DDA measurements were performed on the urine samples. The DDA spectra were searched with the MaxQuant against the UniProt Human Database (December 2014, 88,657 entries) and the indexed retention time standard peptide sequence. A spectral library was generated using the spectral library generation feature in Spectronaut 10. The DIA data from individual samples were analyzed with Spectronaut 10 (Biognosys, Schlieren, Switzerland). First, we converted the DIA raw files into the HTRMS format using the GTRMS converter tool provided with Spectronaut. The FDR was estimated with the mProphet^33^ approach and set to 1% at the peptide precursor and protein levels. The proteins were inferred by the software, and the quantification information was acquired at the protein level using the q-value <0.01 criterion, which was used for the subsequent analyses.

### Study approval

The Institutional Review Board at Seoul National University Children’s Hospital approved the study, and written informed consent was obtained from the parents of participants prior to inclusion in the study. The research was performed in accordance with the Declaration of Helsinki (ethical principles for medical research involving human subjects). This trial has been registered at www.clinicalTrials.gov (NCT02743273).

### Statistical analyses

Normally distributed continuous variables were compared using Student’s t-test or Fisher’s exact test. Nonnormally distributed continuous variables were analyzed using the Mann-Whitney U-test. Paired t tests and x^2^ tests were conducted to assess differences in laboratory measurements between the AKI and control groups. The results are expressed as the means with 95% CIs. Multiple logistic regression was conducted with the candidate biomarkers for the diagnosis of AKI after adjusting for gestational age. In the validation set, biomarkers were dichotomized at the values that optimized the sensitivity and specificity. To assess the predictive accuracy of the candidate biomarkers for the diagnosis of AKI, ROC curves were generated, and AUCs were computed with their CIs. All tests were 2-sided, with p <0.05 indicating statistical significance. All statistical analyses were performed with the IBM SPSS Statistics software package, version 24.0 (IBM, Armonk, NY).

## Supplementary information

Supplementary information.

Supplementary information2.

## Data Availability

The datasets generated during and/or analyzed during the current study are available from the corresponding author on reasonable request.
