# Supplementary material for: Proteomic identification of early urinary-biomarkers of acute kidney injury in preterm infants
Source: Sci Rep. 2020 Mar 4;10:4057. doi: 10.1038/s41598-020-60890-x (PMC7055268; doi:10.1038/s41598-020-60890-x)

**Title**

Proteomic identification of early urinary-biomarkers of acute kidney injury in preterm infants

Young Hwa Jung, MD^2,3^, Dohyun Han, PhD^4^, Seung Han Shin, MD^1,2^, Ee-Kyung Kim, MD, PhD^1,2^, Han-Suk Kim, MD*, PhD^1,2^

^1^Department of Pediatrics, Seoul National University Children’s Hospital, Seoul, South Korea

^2^Seoul National University College of Medicine, Seoul, South Korea

^3^Department of Pediatrics, Seoul National University Bundang Hospital, Seongnam-si, South Korea

^4^Proteomics core facility, Biomedical Research Institute, Seoul National University Hospital, Seoul, South Korea

**Supplementary figures**

**Supplementary Figure S1**. The distribution of the discovered proteins according to the involved cellular components.


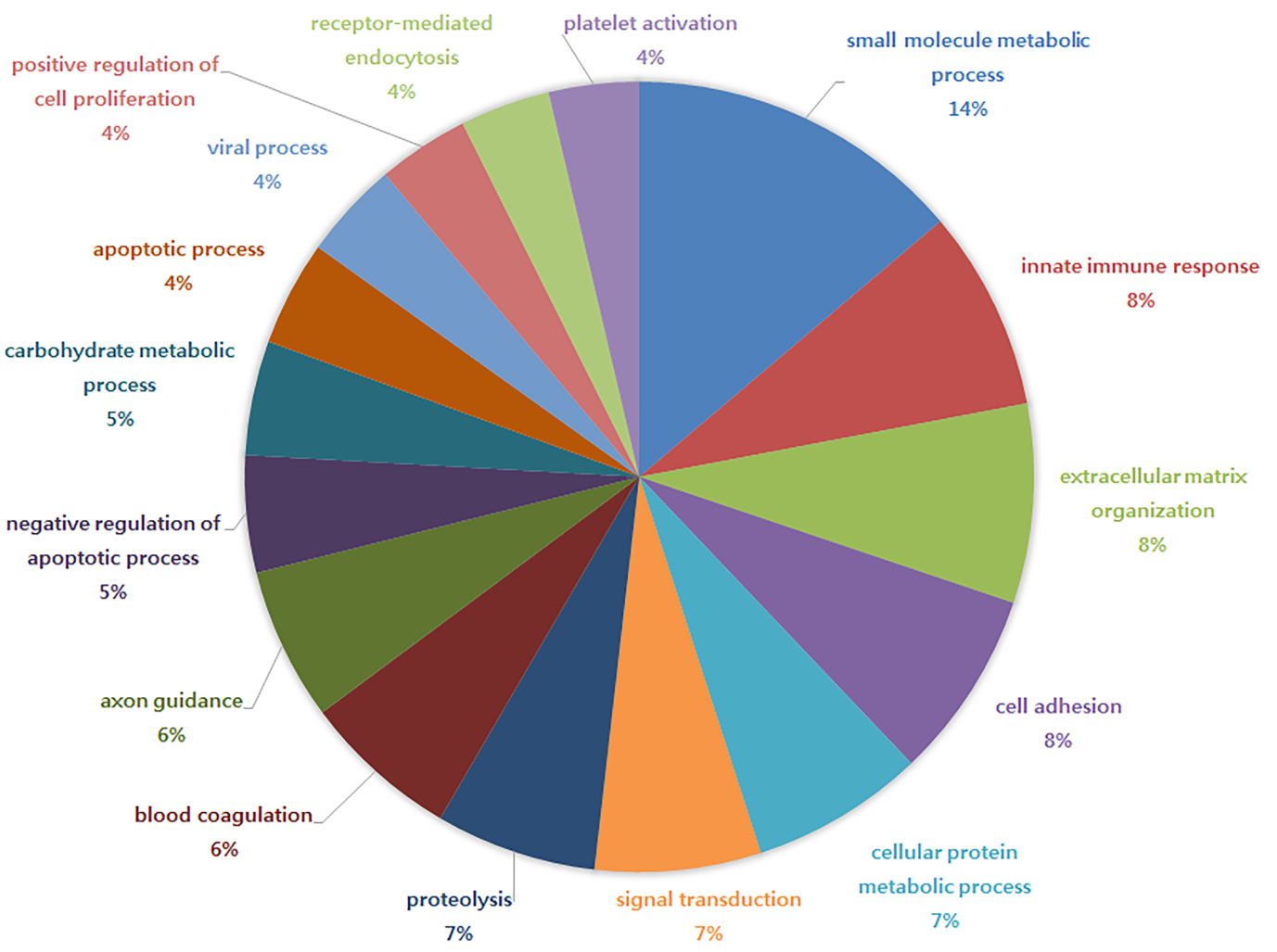


**Supplementary Figure S2.** The distribution of the discovered proteins according to the biological processes.


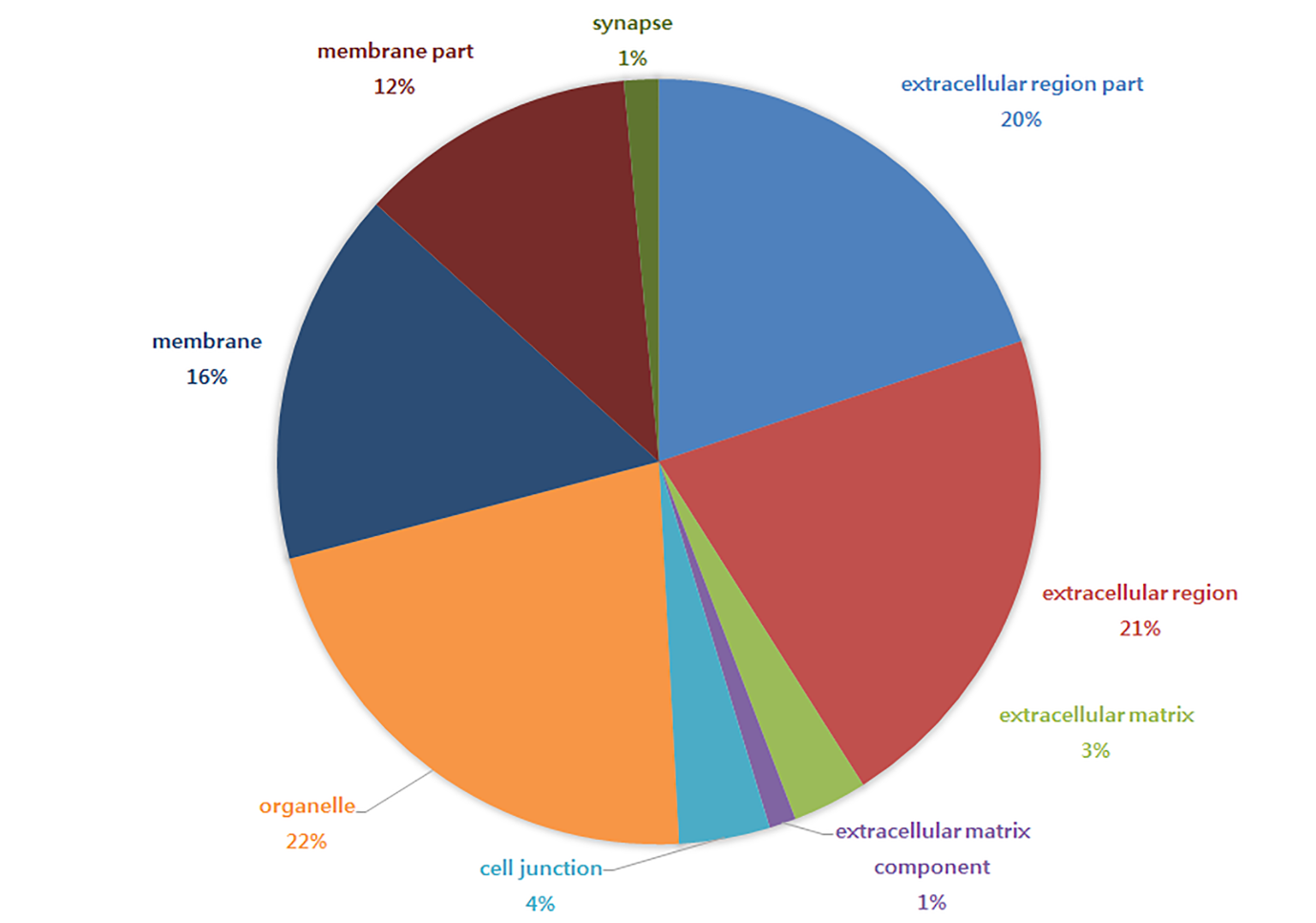

Supplement: Supplementary file 2 — Supplementary information2. [file 41598_2020_60890_MOESM2_ESM.docx]
